# Draft Genome Sequence of Novosphingobium sp. Strain HII-3, a Bacterium Capable of Degrading the Cembranoid α(β)-2,7,11-Cembratriene-4,6-Diol to Farnesal

**DOI:** 10.1128/genomeA.00136-18

**Published:** 2018-04-26

**Authors:** Shen Huang, Yumei Qian, Tao Wei, Chunxiao Jia, Pengfei Yang, Duobin Mao

**Affiliations:** a College of Food and Biological Engineering, Zhengzhou University of Light Industry, Henan, China

## Abstract

Novosphingobium sp. HII-3, the first bacterium confirmed to degrade the cembranoid α(β)-2,7,11-cembratriene-4,6-diol to farnesal, was isolated from cured tobacco leaf in Henan, China. Here, we report the annotated draft genome sequence of strain HII-3, which has an estimated size of 4.45 Mb and comprises 4,072 coding sequences.

## GENOME ANNOUNCEMENT

Cembranoids are 14-carbon cembrane ring cyclic diterpenoids (1). They are distributed primarily in the genera Nicotiana and Pinus and in some marine organisms (e.g., soft coral), in which they are thought to play a key role in survival (2–4). Cembranoids have many important bioactivities, including antimicrobial, antitumor, and neuroprotective activities (5–7). In some circumstances, cembranoids can be converted to solanone, which can be further transformed into useful flavor compounds, such as norsolandione, solanofuran, solanic acid, and others (8). α(β)-2,7,11-Cembratriene-4,6-diol is the largest and most important type of cembranoid.

Genomic DNA from Novosphingobium sp. strain HII-3 was isolated and then used to generate 670 Mb of data using 150-bp paired-end chemistry and an Illumina NextSeq 500 sequencer. Sequence assembly was carried out using SPAdes (http://cab.spbu.ru/software/spades/). The total assembled genome was 4.0 Mb in size and consisted of 90 scaffolds, with an *N*_50_ value of 142,885 bp and an average length of 44,446 bp. The genome was estimated to have an overall G+C content of 64.7%. The draft genome sequence of strain HII-3 was annotated by Prokka, revealing 4,072 protein-coding sequences, with gene lengths ranging from 74 to 6,291 bp. Blast2Go was used to annotate the predicted genes.

Phylogenetic analysis showed that within the Novosphingobium genus, strain HII-3 clustered with N. panipatense strains P5:ABC (9) and SM16^T^ (10), which were isolated from hydrocarbon-contaminated and oil-contaminated soils, respectively. The genome sequence of Novosphingobium sp. HII-3 will provide further insight into the diversity and mechanisms of α(β)-2,7,11-cembratriene-4,6-diol degradation in the environment. Most enzymes responsible for the degradation of cembranoids are membrane proteins. Sequence analysis of the genome of strain HII-3 revealed the presence of several genes coding for cembranoid-degrading and related proteins, namely, carbon-carbon double-bond isomerase, dehydratase, dioxygenases, and monooxygenase. This knowledge of the genes responsible for α(β)-2,7,11-cembratriene-4,6-diol degradation might help to improve our understanding of the biotransformation of cembranoids to other flavor compounds.

### Accession number(s).

This whole-genome shotgun project has been deposited at DDBJ/ENA/GenBank under the accession number PPSM00000000. The version described in this paper is the first version, PPSM01000000.
